# Regulation of Chromatin Acetylation by Alcohol: Dependence on Sex, Brain Region, and Mode of Exposure

**DOI:** 10.3390/genes17060637

**Published:** 2026-05-30

**Authors:** Kelly M. Abshire, Andrey E. Ryabinin, Deena M. Walker

**Affiliations:** 1 Department of Behavioral Neuroscience, Oregon Health & Science University, 3181 S.W. Sam Jackson Park Rd., Mail Code: L470, Portland, OR 97239, USA; abshirek@ohsu.edu; 2 School of Medicine, Oregon Health and Science University, 3181 S.W. Sam Jackson Park Rd., Portland, OR 97239, USA

**Keywords:** alcohol, epigenetics, histone acetylation

## Abstract

Both genetic and epigenetic factors influence the development and pathology of alcohol use disorder (AUD), which is further associated with changes in learning, memory, and synaptic plasticity. Histone acetylation is an epigenetic mechanism that changes the chromatin architecture, influencing gene transcription, which may further impact neuronal signaling, cognition, and addiction-related behaviors. In this review, we summarize the existing literature on how alcohol exposure impacts histone acetylation and the expression and activity of histone acetyltransferases (HATs) and histone deacetylases (HDACs). Overall, alcohol appears to dynamically regulate histone acetylation and the expression and activity of HATs and HDACs in a brain region-, alcohol quantity-, exposure paradigm-, and sex-specific manner. While general patterns exist, more work is needed to elucidate the precise mechanisms through which alcohol changes histone marks across a variety of experimental and biological conditions, thus changing downstream gene expression. We suggest here that a more nuanced understanding of the relationship between histone acetylation and alcohol consumption is needed. Going forward, unbiased molecular techniques for profiling histone marks across the genome will allow for greater precision in determining the impact of alcohol on epigenetic regulation of transcription. However, these approaches must be performed with consideration to differences in mode and quantity of alcohol exposure, as well as withdrawal time and sex, in order for this research to uncover therapeutic targets for future treatment options. Overall, comprehensive, unbiased studies may yield novel insights into the regulatory role of alcohol-induced epigenetic modifications in the pathophysiology and neuropsychiatric correlates of AUD.

## 1. Introduction

Alcohol use disorder (AUD) is a chronic, relapsing psychiatric disease that impacts around 17 million men and 12 million women over 12 years of age annually in the United States [1]. Genome-wide association studies, as well as twin and adoption studies, confirm AUD to be polygenic and have estimated the heritability of approximately 50% [2,3,4]. While these estimates highlight the importance of genetic predisposition, they also indicate the tremendous role of environmental factors contributing to the development of AUD. Indeed, exposure to alcohol itself is an environmental factor and can lead to long-term changes in affective, cognitive, and social behaviors, as well as physiological functions that are, in turn, dependent on age, sex, life history, and amount, length, and pattern of alcohol exposure. Changes in reward-related processes, negative reinforcement, impulsivity, learning and memory, as well as anxiety and depression involved in AUD are mediated by changes in synaptic structure and function. Long-term changes in synaptic physiology, or synaptic plasticity, depend on epigenetic changes regulating gene expression.

Epigenome-wide studies have expanded our understanding of how genes and the environment interact and induce changes in neurobiological processes and physiology in AUD. Epigenetic factors impact gene transcription through mechanisms involving DNA methylation, histone modifications, transcription factors, and noncoding RNA. AUD has been observed to impact epigenetic factors and induce transcriptional changes in systems throughout the body [5]. In the brain specifically, these epigenetic changes appear to have some regional specificity and potential sex differences [6,7,8]. More recent evidence suggests that these AUD-related epigenetic and transcriptional changes have behavioral consequences, which exemplify the power of alcohol exposure as a potent transcriptional modifier while also introducing novel targets for therapeutic intervention for AUD [9]. Since covering all epigenetic mechanisms potentially regulating AUD-related neuroplasticity would be a colossal undertaking beyond the scope of a single review, here, we attempt to focus on alcohol’s effects on the function of histone modifications, specifically histone acetylation, which, in turn, regulates gene transcription and synaptic plasticity.

DNA is condensed into the nucleus of the cell in a highly organized and compact manner, referred to as chromatin. The functional unit of chromatin, the nucleosome, is composed of ~147 base pairs wrapped around core histone octamers consisting of two copies of each of the following proteins: H2A, H2B, H3, and H4. Each histone protein can undergo numerous post-translational modifications (PTMs) in which different functional groups are covalently added to amino acid residues of N-terminal tails—e.g., acetylation, methylation, phosphorylation, ubiquitination, SUMOylation, citrullination, ADP-ribosylation, serotonylation, and dopaminylation, among others. These modifications not only alter the structure of the nucleosome but also change the interaction of DNA with the associated histones, thus increasing or decreasing the likelihood of transcription at a given locus [10]. Histone modifications are diverse, and we are only beginning to understand how the various combinations of PTMs influence or indicate specific transcriptional states [11]. Additionally, new PTMs are being discovered, and novel modified amino acid residues are identified regularly [12]. While a number of histone modifications exist, acetylation of histones, in particular, is a likely candidate for epigenetic modifications being modulated by the effects of alcohol. In fact, recent evidence suggests that the metabolism of alcohol directly contributes to histone acetylation via the deposition of acetyl groups derived from alcohol directly onto the histones [13]. While it is thought that a myriad of histone modifications and their interactions are likely involved in the regulation of alcohol-related behaviors, including consumption, binge drinking, and AUD, histone acetylation is currently one of the more extensively studied PTMs in the AUD field and will be the focus of this review.

Histone acetylation, in general, is a ubiquitous indicator of open chromatin and increased transcriptional potential. Since DNA is a negatively charged molecule, acetylation of histone tails induces an “open” chromatin state by disrupting the associations between the negatively charged DNA and the positively charged lysine residues of the histone tails [14,15,16]. Histone acetylation, especially in response to alcohol, is one of the most studied modifications in epigenetic models of alcohol use disorder [for early reviews see [17,18,19,20]]. While the effects of alcohol intake/exposure are varied, patterns are emerging that help shed light on how alcohol might disrupt the epigenome at both loci-specific and global levels to influence the behavioral correlates of AUD (Table 1).

The following is an overview of the effects of alcohol exposure on histone acetylation at specific lysine residues, as well as a discussion of the effects of alcohol on expression and activity of the “writers” and “erasers”, histone acetyl transferases (HATs) and histone deacetylases (HDACs), across brain regions, dosing paradigms, and mammalian species. This review included PubMed searches with keywords such as “alcohol”, “epigenetic”, “histone”, and others, as well as references found in review papers and in papers identified through the initially performed searches. Preprints were excluded from references. When available, we provide discussion on the potential sex differences that have been observed, but it should be noted that very few studies have investigated these effects in females, highlighting the need for more research on the molecular effects of alcohol exposure in females. We have also contextualized these findings in how they impact factors related to synaptic plasticity, as it is intrinsically linked to the physiology and psychology of AUD.

## 2. Chronic Alcohol Exposure Induces Acetylation of Total H3 and H4 in the Reward Circuitry of Males

Overall, exposure to addictive substances, including alcohol, cocaine, and morphine, has been shown to increase global levels of the histone proteins H3 and H4 acetylation across multiple brain regions [49]. However, the intricacies of how alcohol impacts histone acetylation in various brain regions, species, ages, and exposure paradigms are less understood. In general, repeated exposure to alcohol has region-specific effects on global (i.e., total cellular levels) acetylation at histone proteins H3 and/or H4 throughout the reward circuitry of males. To our knowledge, only one study has investigated the acute (single exposure) effect of alcohol on bulk chromatin acetylation, and they found no changes in the acetylation levels of H2A, H2B, H3, or H4 in the striatum of male mice [31]. However, chronic alcohol exposure (see Table 1 for details on the route and dose) increased acetylation of H3 in the lower midbrain (including the VTA) [48] but not the dentate gyrus and CA3 regions of the hippocampus [46]. On the other hand, global H4 acetylation was enhanced in the lower midbrain [48] and dentate gyrus, unchanged in the CA3 region of the hippocampus [46], and decreased in the nucleus accumbens (NAc) [34].

These global changes are also associated with enhancements of H3ac, but not H4ac, in genes related to synaptic plasticity in the lower midbrain [48]. Similarly, in the prefrontal cortex (PFC), a crucial region for decision making and higher-order processing, chronic injections of ethanol (3 g/kg) were observed to increase H3ac and decrease H4ac at the promoter of the immediate early gene *Fos* and the growth factor brain-derived neurotrophic factor (*Bdnf*) in male rats [24]. Interestingly, these effects appear to be age, sex, and species specific, as the same group reported that chronic intermittent ethanol exposure in *adolescent* male rats and female mice has the opposite effect, in that H4ac is increased at the promoters of *Fos*, *Bdnf*, and *Cdk5*, another key regulator of synaptic plasticity [24,26]. These findings exemplify how age of exposure plays a critical role in determining the impact of alcohol on the epigenome and long-term gene expression, particularly in genes critical for synaptic plasticity. Similar but more complicated effects are observed at the *Bdnf* gene in the hippocampus of the mouse, where 21 days of oral voluntary alcohol consumption increased acetylation of H3 at the *Bdnf* promoter PVI but decreased H3ac at the PIII promoter [46]. The *Bdnf* gene has at least nine promoters, which are thought to be important in activity/stimuli-dependent transcriptional responses [50]. Therefore, these effects may be indicative of alcohol’s influence on stimulus-dependent transcription in the PFC and hippocampus. Together, these data suggest that chronic alcohol exposure influences acetylation of total H3 and H4 in an age-, region- and loci-specific manner and highlights the need for more unbiased and refined approaches (e.g., assessment of specific lysine residues) to investigate the effects of alcohol on histone acetylation.

### 2.1. H3K9ac Is Sensitive to Alcohol Exposure, Particularly in the Amygdala

It is well known that specific lysine residues on H3 and H4 histone proteins are critical for modulating different aspects of transcriptional regulation. H3K9ac (acetylation of the lysine residue at position 9 of the H3) is typically located in the region surrounding the transcription start site [51], and is generally associated with open chromatin with high transcriptional potential [52]. H3K9ac is among the most reported on in the alcohol field, and emerging evidence, particularly in the amygdala, suggests that alcohol exposure results in region-specific alterations to H3K9ac, depending on the length of exposure (acute vs. chronic) and if an animal is in withdrawal (Table 1).

The amygdala is a region known for the regulation of emotional valence and is critical for the mediation of stress- and anxiety-related behaviors tied to addiction and withdrawal in rodents and humans [53,54,55,56]. Therefore, it is not surprising that many studies investigating H3K9ac have focused on the amygdala. Multiple studies have concluded that both acute (1 day) and chronic (weeks to months) alcohol exposure increased global H3K9ac in the amygdala of male rats, regardless of the route of administration (Table 1; [27,39,40,41,42,43]), suggesting that alcohol increases transcriptional potential in this brain region. However, this effect may also be dependent on the length of withdrawal, as H3K9ac was decreased in the amygdala of male rats after 24 h of withdrawal from a chronic ethanol diet [40]. Of note, one study using male mice that had discontinued access to ethanol for 15 days following many months of drinking in a two-bottle-choice (2BC) paradigm found no differences in H3K9ac in the amygdala [29], indicating that withdrawal duration and potentially species contribute to the level of H3K9ac in this brain region.

Similar increases in H3K9ac were observed at the promoter regions of the opioid peptide precursors, prodynorphin and pronociceptin, in the amygdala after acute and chronic intragastric ethanol [43]. On the other hand, H3K9ac/K14ac, two residues that are often co-assessed, were decreased at the *neuropeptide Y* (*NPY*) promoter, a critical regulator of feeding, after chronic oral administration of ethanol in males [36,39]. Both acute and chronic ethanol exposure increased H3K9/K14ac in the promoter regions of important epigenetic regulators such as *Hif3a*, *Creb*, *Creb-binding protein* (*Cbp*), and *p300* [37,38]. These differences in loci-specific effects of ethanol highlight the need for more specific, unbiased approaches, such as Cleavage Under Targets and Release Using Nuclease (CUT&RUN) with sequencing, to fully assess the complex nature of ethanol-mediated epigenetic effects.

It should be noted that even in the amygdala, the global H3K9ac effects of ethanol were subregion-specific and were mostly reported to be increased in the medial (MeA) and central (CeA) but not the basolateral amygdala (BLA) in studies where these regions were separately analyzed [39,40,41,42]; however, one study did not uphold this pattern [27]. Regional selectivity is an important consideration when discussing the underlying biological contributions to AUD vulnerability. For example, the MeA is one of the most well-characterized sex-specific brain regions in the extended mesocorticolimbic reward circuitry. The MeA is 50–80% larger in the male rodent compared to females [57,58] and is critical for sex-specific reward-associated behaviors [59]. The MeA was also identified as a key sex-specific mediator of cocaine reward [60]. The MeA projects to the CeA, the critical “hub” region for the behavioral correlates of AUD in the rodent [61,62,63,64,65]. Together, these subregion-specific effects of ethanol on H3K9ac, combined with the sexually dimorphic nature of the MeA, suggest that transcriptional changes in the MeA—CeA neurocircuit may be a critical mediator of sex-specific alcohol-mediated synaptic plasticity.

While H3K9ac has been most extensively studied in the amygdala, the effects of alcohol on H3K9ac in other brain regions appear to be largely dependent on the length of ethanol exposure (Table 1). For example, a single injection of ethanol had no effect on global H3K9ac/K14ac when analyzed across the entire cerebral hemisphere [22], the cerebral cortex [21], or nucleus accumbens (NAc) [25], but increased global H3K9/K14ac in the hippocampus [13,23]. However, these changes were assessed by Western blot, which may not be sensitive enough to detect important subtle effects of acute ethanol on H3K9ac compared to chromatin immunoprecipitation (ChIP) coupled with qPCR (qChIP) or next-generation sequencing (ChIP-seq). For example, ChIP revealed that acute ethanol decreases H3K9ac at the GABAergic marker, *Gad1*, and the *histone deacetylase 2* (*HDAC2*) in the cerebral cortex [21] and yielded widespread changes, including enhanced H3K9ac presence at key neuronal genes in the hippocampus [13]. These results highlight the need for more studies using unbiased genome-wide approaches to identify alcohol-sensitive loci throughout relevant circuitry.

These region-specific effects were also observed with chronic ethanol exposure and appeared to be dependent on the length of withdrawal. In subregions of the PFC [23,27,28,30,66] and NAc [28], chronic ethanol exposure increased global and gene-specific H3K9ac (Table 1) within the first few days following ethanol exposure. However, H3K9ac seemed to return to control levels following prolonged withdrawal in the PFC. In the hippocampus, H3K9ac was diminished only after 24 h of withdrawal [47], and while there are contradictory reports in the NAc, these are likely due to differences in age of exposure and ethanol having a distinct impact on H3K9ac in its core versus shell subregions [23,25,28]. Together, these region-specific effects of acute and chronic ethanol on H3K9ac provide intriguing insight into the role each region may play in the progression of AUD4.

### 2.2. Alcohol’s Effects on H3K14ac

Similarly to H3K9ac, H3K14ac is also considered to be a marker of chromatin accessibility and a target for transcription factors to bind to DNA [52,67]. As mentioned above, few studies have probed H3K14ac independently because of its high rate of co-occurrence with H3K9ac [68]. Those who have investigated specific changes in H3K14ac have identified variations depending on exposure time and brain region. For example, chronic exposure to ethanol vapor, but not withdrawal, increased H3K14ac in the NAc of adult male mice [28]. However, withdrawal from chronic ethanol consumption decreased H3K14ac in the basal forebrain and lateral hypothalamus [35]. No changes were identified in the PFC following chronic ethanol vapor or withdrawal [28]. Though H3K14ac remains understudied in AUD, it has a known importance in long-term memory and regulation of *Creb* gene expression [69], representing a critical potential role in regulating alcohol-related changes in synaptic plasticity.

### 2.3. Alcohol’s Effects on H3K27ac

Although less is known about the transcriptional regulatory mechanisms of H3K27ac compared to other histone marks, it is typically enriched in the promoter region and is considered a marker of active enhancers [51]. Very few studies have investigated the relationship between global H3K27ac and alcohol exposure. Mews et al. identified enhanced global and gene-specific levels of H3K27ac in the hippocampus in response to acute ethanol administration in male mice [13]. In the NAc, H3K27ac levels were increased following chronic oral consumption and at least 12 h of withdrawal, but no changes were identified in animals with only two days of alcohol exposure [33]. Lastly, no differences in global H3K27ac levels were identified in the PFC following chronic ethanol vapor exposure or withdrawal in male mice [28]. While understudied, this enhancement of H3K27ac is in line with other literature suggesting an overall increase in H3ac, and presumably open chromatin and gene transcription, in response to alcohol exposure.

Gene-specific effects have been investigated in the amygdala, specifically in the context of its regulatory function of *activity-related cytoskeletal* (*Arc*) gene expression, and these effects seem to depend on the length of exposure. Here, acute ethanol increased H3K27ac occupancy at the *Arc* Synaptic Activity-Responsive Element (SARE) site and promoter, whereas chronic ethanol decreased H3K27ac, and giving chronically exposed rats an acute dose of ethanol returned it to levels of saline-treated control in male animals [44]. Interestingly, this observation seems to be at least partially recapitulated in humans, as postmortem amygdala tissue from a mostly male sample showed a decrease in H3K27ac occupancy at the *Arc* SARE site in AUD subjects compared to non-AUD controls [45]. While sex differences were not investigated in these studies, others have observed sex differences in *Arc* expression at baseline in the hippocampus, which is alleviated in response to stress [70], suggesting that ARC may be an important sex-specific target of stimuli such as ethanol. This hypothesis deserves follow-up studies.

Together, these data suggest that alcohol has impacts on H3ac that are brain region-, age-, gene-, and length of ethanol exposure-specific. Many of the gene-specific changes in H3ac were on genes such as *Arc*, *Bdnf*, and *Npy*, which are all key regulators of synaptic transmission and are critical in learning-related plasticity. Further research is needed to understand how alcohol-induced global and gene-specific changes in H3ac contribute to the neurobiology of the disorder and its behavioral correlates.

### 2.4. Alcohol’s Effects on H4ac

The impact of alcohol on H4 acetylation overall, and at specific lysine residues, is less investigated compared to H3. To date, the available results suggest that the relationship between the effects of alcohol and global H4 acetylation is complicated and varies across experimental paradigms, which involve differences in age, brain region, and withdrawal status. For example, chronic intraperitoneal ethanol administration in male rats and female adolescent mice increased H4ac at specific genes (see Table 1 for details) in the PFC, but decreased H4ac when male rats were exposed to alcohol in adulthood [24,26]. Chronic oral ethanol consumption increased global H4ac levels in the hippocampus of male mice [46], but decreased H4ac in the NAc of male mice and rats [34]. In the lower midbrain, global H4ac increased in adult outbred ddY mice made dependent on alcohol through chronic exposure to vapor, as well as in those experiencing acute withdrawal following this exposure [48]. No changes were observed in the striatum following a single exposure in male mice [31]. Therefore, further studies are needed to parse through the intricacies of the relationship between H4ac, ethanol consumption, and animal and experimental characteristics.

### 2.5. Alcohol’s Effects on H4K8ac

H4K8ac is typically enriched at the promoter and actively transcribed regions of genes [51]. In addition to its role in facilitating an open chromatin state, H4K8ac is also known to participate in the recruitment of transcription complexes, including RNA Pol II [67]. Few studies have investigated H4K8ac in relation to the effects of alcohol, and all are limited to the amygdala of male rats. Overall, they find that both acute and chronic ethanol exposure increased global H4K8ac in the CeA and MeA, but not BLA, of adult rats [39,40,41]. However, these effects may be dependent on the presence of alcohol itself, as H4K8ac decreased in animals that were 24 h into withdrawal following a chronic ethanol diet [40]. As mentioned earlier, the MeA is a known sexually dimorphic brain region, and these changes may be sex-specific based on the fact that this region is critical for sex differences in both naturally [59] and drug-related rewarding behaviors [60].

### 2.6. Alcohol’s Effects on H4K12ac

Akin to H4K8, H4K12ac is enriched around promoters and transcribed regions of active genes [51]. Changes in H4K12ac in response to alcohol appear to be dependent on the brain region and age of exposure. More specifically, adolescent rats appear to be more vulnerable to alcohol-induced H4K12ac changes, with this mark being increased in the frontal cortex and NAc and decreased in the striatum in adolescent but not adult males that were chronically exposed to ethanol [23]. However, a study in female mice showed that chronic alcohol exposure increased H4K12ac in the PFC 24 h after the last alcohol injection, but this enhancement was no longer present after 3 weeks of no alcohol [26]. Another study using female mice found no differences in H4K12ac following acute and chronic alcohol exposure in female mice in the dorso-lateral and dorso-medial striatum 30 min after final exposure [32]. Interestingly, this group also identified an increase in H4K12ac in the NAc core, but not shell, following acute and chronic alcohol [71]. While the number of studies is few, together they indicate alcohol may differentially regulate H4K12ac depending on age, length of alcohol exposure, and withdrawal.

Taken together, acute and chronic alcohol exposure appear to generally increase H3 and H4 acetylation, both globally and in a gene-specific manner across multiple brain regions and in multiple rodent species, ages, and alcohol exposures. Some studies indicated that alcohol withdrawal, underlying sensitivity to alcohol, and related behavioral output may also be linked to histone acetylation. Unfortunately, differences in experimental design, route of alcohol exposure, and time since last exposure make it difficult to form concrete conclusions on alcohol’s impact on histone acetylation in the brain and its impacts on behavior and synaptic plasticity therein. Other limitations include a lack of exploration in brain regions outside of the traditional reward circuitry structures, such as the hypothalamus, thalamus, cerebellum, and sensory brain areas, all of which are likely to be impacted by alcohol consumption.

## 3. Alcohol’s Effects on Histone Acetyltransferases and Deacetylases

Histone-modifying enzymes are responsible for the addition (writers) and removal (erasers) of chromatin modifications within the nucleus. With regard to histone acetylation, the expression (both mRNA and protein) and enzymatic activity of these histone acetyltransferases (HATs) or histone deacetylases (HDACs) are impacted by stimuli, which, in turn, add or remove acetyl groups from a given histone protein. While the interactions of these enzymes with histone targets are complex and we are only beginning to fully understand the extensive roles they play in gene regulation, emerging evidence suggests that each enzyme has specific targets in the cell, cytoplasmic or nuclear, which dictate how environmental stimuli influence transcription. Here, we will discuss the known impacts of alcohol on the expression and activity of HATs and HDACS and how this may influence the transcriptional profiles induced by alcohol throughout the brain.

### 3.1. Acute and Chronic Alcohol Effects on HAT Activity Are Region Specific

HATs are a group of enzymes that transfer the acetyl group from acetyl-CoA to lysine residues on histones. The exact catalytic mechanisms behind this transfer of acetyl groups and substrate binding specificity vary between the HAT subfamilies [72], which leads to slightly different roles in biological processes and pathology [73,74]. The extent to which HATs are involved in alcohol-induced changes in transcription is still being uncovered. There is evidence that enzymatic activity induced by ethanol is region specific (Table 2). In the amygdala of female mice, acute and chronic systemic alcohol injections enhanced the activity of HATs, as measured by calorimetric activity, within the nucleus but decreased it in the cytosol (with acute treatment only) [32]. In contrast, HAT activity is increased in the cytosol, and not the nucleus, of cells in the striatum following acute and chronic alcohol injections [32]. These differences in HAT activity in the cytosol versus the nucleus indicate potential differences in the specific HAT protein affected, as well as the protein targets for acetylation and biological outcomes. An increase in nuclear HAT activity likely reflects an increase in chromatin acetylation and gene transcription, while cytoplasmic HAT activity may involve the acetylation of other proteins to aid in shuttling to the nucleus or that may regulate the cytoskeleton, cellular transport, and ion channel function [75]. Furthermore, there were no changes in HAT activity in the hippocampus or PFC [24,32], indicating that alcohol-induced changes in HAT activity are brain region specific. Age and potentially sex may also play a role in differential HAT activity. Chronic alcohol injections increased HAT activity in the PFC of adolescent but not adult male rats 24 h after the last injection. However, this effect does not seem to be persistent, as no changes were observed 2 weeks after the last alcohol exposure at either age [24]. These results highlight the complexity by which alcohol influences acetyltransferase activity within the cell and across brain regions, sexes, and ages. Therefore, there is clearly much more to discover about the specific role HATs play in mediating alcohol’s molecular and cellular consequences across the nervous system.

There is a diversity of HATs expressed in the brain, and we are only beginning to decipher their roles in modulating alcohol reward. Here, we have chosen to focus on those expressly linked to changes in synaptic plasticity and reported on in the context of alcohol-related behaviors. The cAMP-response element binding protein (CREB) is one of the commonly studied transcription factors in synaptic plasticity, particularly with regard to substance and alcohol use disorders [76,77]. It acts by binding to the cAMP response element (CRE) at gene promoters, where it is then phosphorylated by receptor-activated protein kinases at Ser133 [78]. Phosphorylated CREB (pCREB) is a transcription factor and has been implicated in the pathology of psychiatric disorders [79,80,81], likely through its known regulatory functions of neuronally relevant genes such as *Bdnf* [82]. The influence of alcohol on pCREB in the brain appears to be region-dependent. In the amygdala, pCREB levels were decreased in the CeA and MeA following 24 h of withdrawal from a chronic alcohol diet and more than a month after repeated adolescent alcohol exposure, but not while rats were actively on an alcohol diet [38,83]. In the PFC of female mice, pCREB was increased 24 h after the last exposure to a series of chronic intraperitoneal alcohol injections, but pCREB was decreased 3 weeks later [26], further exemplifying the importance of the length since last alcohol exposure on pCREB levels and variation across different brain regions. In the striatum of male rats, pCREB levels increased after a single injection of ethanol, did not change following a chronic ethanol diet compared to pair-fed controls, and were reduced in chronically alcohol-fed rats compared to pair-fed controls when both were given in an acute ethanol challenge [84]. Conversely, in the lower midbrain of ddY mice, chronic exposure to ethanol vapor chambers and withdrawal from this chronic exposure increased pCREB [48]. A different pattern was observed in the cerebellum, where an acute injection of ethanol increased pCREB in both ethanol naïve male rats and those chronically exposed through diet. However, acute exposure was significantly more stimulating in alcohol naïve rats, while a chronic ethanol diet without an acute IP exposure decreased pCREB compared to pair-fed controls, suggesting that this effect is modulated by alcohol dependence [85,86]. Overall, it appears that acute alcohol exposure increases pCREB, while chronic exposure has mixed effects depending on the brain region, route of exposure, and potentially sex and species. Considering CREB’s known importance in enhancing synaptic plasticity and neuronal excitability, future research should further probe this relationship between alcohol use and pCREB regulation in a region- and sex-specific manner.

**Table 2:** Alcohol’s effects on histone acetyltransferases.

Brain Region	Marker	Chronic/Acute	Route (Dose)	Withdrawal	Effect	Species (Strain)	Sex	Refs
Cerebral Cortex	Kat2b	Acute (1 dose)	IP (3g/kg)	6 h	mRNA: ↑	Mouse (C57BL/6J)	M	[21]
Prefrontal Cortex	HAT Activity	Acute (1 dose)	IP (2g/kg)	30 min	enzymatic activity: nc (nuclear & cytoplasmic fraction)	Mouse (DBA/2J)	F	[32]
		Chronic (8–10 doses)	IP (2–3 g/kg)	30 min & 24 h	enzymatic activity: nc (nuclear & cytoplasmic fraction)	Mouse (DBA/2J) Rat (Wistar)	F (mice) & M (rats)	[24,32]
	pCREB	Chronic 8 doses	IP (3g/kg)	24 h	Protein: ↑	Mouse (C57BL/6J)	F	[26]
				3 weeks	Protein: ↓			
Striatum	HAT Activity	Acute (1 dose)	IP (2g/kg)	30 min	enzymatic activity: nc (nuclear fraction); ↑ (cytoplasmic fraction)	Mouse (DBA/2J)	F	[32]
		Chronic (10 doses)	IP (2g/kg)	30 min	enzymatic activity: nc (nuclear fraction); ↑ (cytoplasmic fraction)			
	Kat2a	Acute (1 dose)	IP (2g/kg)	30 min	mRNA: nc	Mouse (DBA/2J)	F	[32]
		Chronic (10 doses)	IP (2g/kg)	30 min	mRNA: ↑			
	pCREB	Acute (1 dose)	IP (2.5g/kg)	1 h	Protein: ↑	Rat (Sprague Dawley)	M	[84]
		Chronic (5 wks)	Oral: liquid diet	0 h	Protein: nc			
			Oral: liquid diet + IP challenge	30 min	Protein: ↓			
Amygdala	HAT Activity	Acute (1 dose)	IP (2g/kg)	30 min	enzymatic activity: ↑ (nuclear fraction); nc (cytoplasmic fraction)	Mouse (DBA/2J)	F	[32]
		Chronic (10 doses)	IP (2g/kg)	30 min	enzymatic activity: ↑ (nuclear fraction); ↓ (cytoplasmic fraction)			
	pCREB	Chronic: (Various)	Oral: liquid diet (9% *v*/*v*) or IP (2 g/kg)	1 h	Protein: nc in CeA, MeA, or BLA	Rat (Sprague Dawley)	M	[83]
				24 h	Protein: ↓ in CeA and MeA; nc in BLA	Rat (Sprague Dawley)	M	[83]
				~50 days	Protein: ↓ in CeA and MeA; nc in BLA	Rat (Sprague Dawley)	M	[38]
	CBP	Acute (1 dose)	IP (1–2 g/kg)	1 h	Protein: ↑ in CeA and MeA, not BLA; ↑ at Arc SARE & promoter mRNA: ↑	Rat (Sprague Dawley)	M	[38,40,44,87,88]
		Chronic (Various)	Oral: liquid diet or IP (2 g/kg)	0 h	protein: nc (CeA, MeA & BLA)	Rat (Sprague Dawley)	M	[40]
				1 h	Protein: nc at ARC SARE or promoter; mRNA: ↑	Rat (Sprague Dawley)	M	[38,44]
				+24 h	protein: ↓ (CeA & MeA); nc (BLA)	Rat (Sprague Dawley)	M	[40]
				~50 days	Protein: ↓ at ARC SARE and promoter; mRNA: ↓	Rat (Sprague Dawley)	M	[38,44]
Hippocampus	HAT Activity	Acute (1 dose)	IP (2g/kg)	30 min	enzymatic activity: nc (nuclear & cytoplasmic fraction)	Mouse (DBA/2J)	F	[32]
		Chronic (10 doses)	IP (2g/kg)	30 min	enzymatic activity: nc (nuclear & cytoplasmic fraction)	Mouse (DBA/2J)	F	[32]
	CBP	Chronic (8 doses)	IP (2g/kg)	~50 days	Protein: ↓	Rat (Sprague Dawley)	M	[89]
	Kat2b	Acute (1 dose)	IP (3g/kg)	+6 h	mRNA: nc	Mouse (C57BL/6J)	M	[21]
Lower Midbrain (including VTA)	pCREB	Chronic: (9 days)	Vapor	0 h–3 days	Protein: ↑	Mouse (ddY)	M	[48]
Cerebellum	pCREB	Acute	IP (2.5–3 g/kg)	5–60 min	Protein: ↑	Rat (Sprague Dawley)	M	[85,86]
		Chronic (5 wks)	Oral: liquid diet & IP (2.5 g/kg)	30 min	Protein: ↓	Rat (Sprague Dawley)	M	[86]

Abbreviations: BLA: basolateral amygdala; CBP: Creb binding protein; CeA: central amygdala; F: female; HAT: histone acetyltransferase; Hrs: hours; IP: intraperitoneal; M: male; MeA: medial amygdala; min: minutes; nc: no change; Refs: references; VTA: ventral tegmental area.

Once CREB is phosphorylated, CREB-binding protein (CBP) is then recruited to the activated CREB, and transcription is initiated [90,91]. CBP, often referred to alongside its paralog, p300, has a widespread impact on protein function and gene regulation [91], is autoacetylated [92], and has been reported to specifically target H3K27 for acetylation [93]. This process is critical for learning and memory and is impacted by both chronic and acute ethanol exposure in an age- and region-specific manner. In a series of studies looking at both protein and mRNA, Pandey and colleagues found that in the amygdala, specifically the CeA and MeA, CBP levels increased after acute ethanol and decreased following withdrawal from chronic exposure [38,40,87,88]. However, CBP levels were unchanged while rats were on a chronic alcohol diet, and the reduction in *CBP* transcription associated with withdrawal could be restored to baseline levels following re-exposure to alcohol [38,40]. In addition to the global changes in CBP and mRNA expression, evidence suggests that alcohol can impact the interaction between CBP and cis-regulatory regions of the DNA. Specifically at the *Arc* SARE site and promoter, CBP was increased following an acute ethanol injection, decreased following chronic exposure, and not changed when chronically exposed animals were given an acute ethanol injection compared to saline controls [44]. Arc expression is necessary for processes such as neuronal activity, long-term potentiation, and memory consolidation, all of which are associated with synaptic plasticity [94]. These results imply that CBP is dynamically regulated by alcohol and provide evidence that histone acetylation at H3K27 may be a critical target for alcohol-induced transcriptional regulation, specifically for genes associated with synaptic plasticity. Lastly, chronic ethanol injections during adolescence yielded decreased CBP in the hippocampus of male rats, as measured by gold immunolabeling [89]. Therefore, alcohol’s impact on CBD is likely also age and brain region specific.

Lysine acetyltransferases 2a/2b (Kat2a/2b) preferentially acetylate histone H3, and to a lesser extent, H4, and are part of the Gcn5-related N-acetyltransferase family, which is one of the most explored nuclear HAT subfamilies [95]. Kat2a (also known as GCN5) is primarily known as a transcriptional activator. The Kat2b protein, also known as p300/CBP associating factor (PCAF), is a transcriptional coactivator through its association with CBP/p300 and is thought to specifically acetylate H3K9 [96]. Both Kat2a and Kat2b histone acetylation actions can change the chromatin structure and have been implicated in regulating gene expression, DNA replication and repair, and cell cycle progression [97,98,99,100,101,102,103]. Despite a number of investigations probing the relationship between alcohol exposure and H3K9ac, the functional role of Kat2a/2b remains largely unexplored. However, studies have tried to infer differences in enzymatic activity and histone acetylation by measuring changes in mRNA expression in reward-sensitive brain regions. These effects seem to be sex and region specific. In female mice that were resistant to chronic ethanol-induced behavioral sensitization (EIBS; defined by the authors as hyperlocomotion), there were increased *Kat2a* mRNA levels in the striatum compared to saline controls, but this effect was not seen in EIBS-sensitive animals or acute ethanol-exposed mice [32]. In males, expression of *Kat2b* was increased after a single injection of ethanol in the cerebral cortex but not the hippocampus [21], and chronic exposure to ethanol vapor was associated with increased *Kat2b* mRNA in the NAc but not the PFC [28]. These findings are interesting considering that Kat2a is known to mediate hippocampal synaptic plasticity and long-term memory consolidation [104]; however, Kat2b’s role in these functions remains unclear. While these initial studies indicate both sex and regional specificity for Kat2a/2b, more work is needed to further characterize these relationships.

### 3.2. Alcohol’s Effects on Histone Deacetylases Are Age and Sex Dependent

Counter to HATs, histone deacetylases (HDACs) remove acetyl groups from ε-amino lysines, increasing the positive charge on histones and strengthening the histone–DNA interaction to reduce transcriptional potential. In addition, HDACs may also suppress the transcription of certain genes by deacetylating specific sites so that transcriptional repressors may bind. HDACs and their pharmaceutical inhibitors have been implicated in the pathology and potential therapeutic future of neurodegenerative and neuropsychiatric disorders for decades (for review: [105]). In fact, preclinical studies are also beginning to confirm the promise that HDAC inhibitors hold for treating excessive alcohol use and withdrawal symptoms [34,47]. However, changes in alcohol-induced HDAC activity appear to vary based on sex, brain region, and species (Table 3).

In the amygdala, while no differences in activity have been observed in female rats [32], acute ethanol decreases HDAC activity in male rats. This activity normalized when males were on a chronic ethanol diet and increased in withdrawal [40,41,42]. Sex and age of exposure also seem to play a role, as male rats that were chronically exposed during adolescence displayed decreased HDAC activity in the amygdala during adolescence but increased activity following 24 h of withdrawal or when exposure was stopped, and animals were aged to adulthood [110]. Additionally, adolescent male rats that were chronically given IP ethanol showed increased HDAC activity in adulthood in the hippocampus [89]. In the female but not male PFC, acute and chronic ethanol exposure increased HDAC activity in the nucleus [24,32]. Female mice also showed a shift in HDAC activity in the striatum: lower in the nucleus and higher in the cytosol following both acute and chronic ethanol exposure, though no changes were observed in the hippocampus [32]. These differences in nuclear versus cytoplasmic activity likely indicate discrepancies in which specific HDAC enzymes are impacted by alcohol exposure and withdrawal. In the NAc, no changes in HDAC activity were observed after two alcohol drinking sessions, but HDAC activity was reduced 22 h following 10 days of drinking [33], a finding re-emphasizing the importance of the length/quantity of alcohol exposure and withdrawal. Taken together, there is evidence to suggest brain region-specific sex differences in ethanol-induced changes in HDAC activity. However, differences in experimental methodology, species, and route of ethanol administration, as well as a dearth of studies in some regions and across both sexes, complicate this conclusion.

### 3.3. Alcohol’s Effects on Class I HDACs

There are several different HDAC enzymes, and they are subdivided into different classes. Class 1, characterized by their similarity to the yeast transcription regulator reduced potassium dependency 3 (Rpd3) sequence, consists of HDAC1, HDAC2, HDAC3, and HDAC8. Class I HDACs are generally considered to be nuclear proteins that are ubiquitously expressed (for review: [111]). Studies on alcohol-induced changes in Class I HDACs are limited but do exemplify age- and brain region-specific changes (Table 3).

Acute ethanol exposure increased *Hdac1* gene expression in the amygdala of male rats and dorsal striatum of male and female mice but not the PFC or whole striatum [32,106,107,108]. Conversely, chronic oral consumption decreased *Hdac1* mRNA in the hippocampus [46] but not the PFC of male rats. [107,108]. A more complicated relationship appears to be occurring with HDAC2. First, adolescent ethanol exposure led to increased HDAC2 protein and mRNA levels in the adult male amygdala and following withdrawal in the adolescent amygdala [110]. This increase in *Hdac2* transcription was also observed in the adult male amygdala following a single ethanol exposure [107]. However, this effect may be dependent on the subregions of the amygdala or differences in route of exposure, as others have shown that both acute and chronic ethanol exposure *decreased* HDAC2 protein levels in the CeA and MeA of male rats [39,42]. While no differences in HDAC2 expression were observed in the PFC or NAc [28,106,107,108], HDAC2 was decreased in the dorsal striatum and cerebral cortex of male mice, as well as the striatum of female mice, following acute ethanol exposure [21,32,106,108]. An increase in *Hdac2* mRNA following a chronic ethanol diet, as well as withdrawal, was observed in the hippocampus of adult male rats [46,47], whereas chronic exposure decreased *Hdac2* expression in the striatum [32,106]. However, an important caveat is that these studies were conducted using female mice and male rats, so a species effect cannot be ruled out. Regarding HDAC3, several groups have reported no changes in *Hdac3* transcription in the PFC, hippocampus, striatum, or amygdala following acute or chronic ethanol [32,46,107]. In contrast, in a group of mixed sex mice, acute ethanol exposure decreased HDAC3 levels in the dorsal striatum and PFC [108]. Heterogeneity in sex, species, and route of ethanol administration likely accounts for the differences in reports. Finally, *Hdac8* expression was elevated in the amygdala following chronic, but not acute ethanol exposure in male mice [107]. In contrast, female mice displayed elevated striatal transcription of *Hdac8* following acute but not chronic ethanol exposure [32]. Although Class I HDACs are largely homologous and share a large portion of their amino acid sequence, factors such as age, brain region, dosage, and number of alcohol exposures, and likely sex, seem to differentially regulate each specific HDAC’s response to alcohol. Future studies are needed to characterize these specific relationships.

### 3.4. Alcohol’s Effects on Class II HDACs

The Class II HDAC proteins are HDAC4, HDAC5, HDAC6, HDAC7, HDAC9, and HDAC10, and are grouped together due to their sequence similarity to the yeast Hda-1 protein. Compared to Class I HDACs, Class II proteins likely have some additional extra-nuclear functions and are located in both the nucleus and cytoplasm. More specifically, class IIa HDACs (HDAC4, 5, 7, and 9) harbor a binding site for transcription factor MEF2, and are known to shuttle between the nucleus and cytoplasm in an activity-dependent manner [112]. On the other hand, class IIb HDACs (HDAC6 and 10) are typically found in the cytoplasm, are known to deacetylate proteins such as α-tubulin and cortactin, and are implicated in metabolic function [113]. Moreover, each HDAC isoform likely has its own impact on cerebral function and synaptic plasticity [114]. These differences in function add to the complexity in determining the diverse subcellular effects of ethanol on acetylation. However, evidence suggests ethanol-induced changes in class II HDACs may be dependent on region, sex, age, and length of exposure.

In the PFC, the route of administration may influence alcohol’s impact on *Hdac4*, as expression levels were reduced following chronic intraperitoneal injections but not after acute or chronic oral gavage [106,107]. While no effects of acute exposure were observed, chronic ethanol injections decreased *Hdac4* gene expression in male rats but not in female mice [32,106]. In the ventral striatum (also known as NAc), nuclear HDAC4 protein levels were reduced 18 h after the rats were removed from chronic oral alcohol access, though this effect was not present at 0 or 12 h of withdrawal [33]. Finally, in the amygdala, no changes in HDAC4 or *Hdac6* expression were observed following acute or chronic alcohol exposure in adult male rats [39,42,107]. However, following adolescent ethanol exposure, there was an increase in HDAC4 protein in the amygdala of male rats during withdrawal, but not while animals were drinking, and changes did not persist into adulthood [110].

Studies investigating the regulation of HDAC5 expression by ethanol have produced conflicting results. Following acute exposure to ethanol, *Hdac5* transcription was elevated in the amygdala of male rats [107] but not in the PFC or striatum of male and female mice [32]. An even more complicated picture emerges with chronic treatments; while some have found that chronic ethanol decreased *Hdac5* mRNA in the PFC, striatum, and hippocampus in male mice [46,106], others have shown no effects of chronic ethanol in the PFC or striatum of male and female mice [32,107] or the amygdala of male rats [107].

Expression of *Hdac7* mRNA was upregulated in the striatum following chronic ethanol exposure in female mice, but no changes were seen following acute ethanol or in the amygdala or PFC of male rats [32,107]. Furthermore, female mice had elevated *Hdac9* expression in the striatum following acute but not chronic ethanol exposure [32]. However, no changes in *Hdac9* were observed in the amygdala, hippocampus, or PFC of male mice after acute or chronic ethanol [46,107]. Lastly, female mice that were chronically exposed to ethanol showed decreased *Hdac10* expression in the striatum, while male rats showed increased expression in the PFC after a single dose of ethanol [32,107]. Taken together, Class II HDACs are impacted by alcohol in a dose-dependent, brain region-specific, likely sex-specific manner, but the lack of studies on these enzymes precludes any broad conclusions from being made. Given that class IIa HDACs, in particular, are also known to play a role in neuronal growth, cell signaling, and neuronal toxicity [115], these enzymes represent important future targets for the alcohol and neurocognitive fields alike.

### 3.5. Alcohol’s Effects on Class IV HDACs

HDAC11 is the only Class IV HDAC protein and has a sequence similar to both Class I and II HDACs. HDAC11 is considerably less understood than most other HDAC proteins, but has been identified as a regulator of DNA replication factors [116]. To our knowledge, HDAC11 protein levels have not been explored in relation to alcohol exposure, but a few studies have investigated differences in alcohol-induced gene expression. First, a single dose of ethanol decreased *Hdac11* gene expression in the cerebral cortex of male mice [21]. In contrast, no other differences in *Hdac11* expression have been observed in the hippocampus, amygdala, or PFC in male mice following acute or chronic ethanol exposure [21,28,107]. However, *Hdac11* expression does seem to be sensitive to ethanol exposure in a sex- and subregion-specific manner. In female mice, both acute and chronic ethanol exposure decreased *Hdac11* gene expression in the striatum [32]. Additionally, in the NAc/ventral striatum, high-ethanol-drinking mice showed increased levels of *Hdac11* mRNA expression compared to their low-drinking counterparts [109]. However, another study using ethanol vapor and intraperitoneal injections did not observe the same effect [28], which could be due to differences in study design, withdrawal time, and route of administration. Given HDAC11’s role in DNA replication and the known cytotoxic effects of alcohol, future studies should explore how alcohol induces changes in HDAC11 expression and activity across different brain regions, with consideration given to sex differences, which are underexplored and thus not readily comparable for this enzyme.

## 4. Conclusions and Future Directions

Several conclusions can be drawn from the studies presented. First, the field of research exploring the epigenetic underpinnings of AUD and alcohol consumption is quickly expanding and has already made impressive progress in connecting alcohol-related changes in the chromatin landscape to neuronal function and addiction-related behaviors; however, there is still ample need for more work. Given the recent evidence showing that alcohol can directly contribute acetyl groups to histones [13], it is no surprise that most studies report an increase in H3 and H4 acetylation across species and alcohol consumption paradigms. In fact, most studies indicated a decrease in histone acetylation measured levels following a period of withdrawal, exemplifying how these processes are dynamic and shift quickly in reaction to environmental changes. While limited in the number of studies, the work on HATs and HDACs offers further insight into this process. In general, HATs, specifically pCREB and CBP, increase after acute exposure and decrease following chronic exposure. Regulation of HDACs by alcohol has proven to be much more complicated and specific to the particular HDAC protein, brain region, and degree of alcohol exposure. Depending on the involved brain region and mode of exposure, the alcohol-mediated modifications of histones likely contribute to transcriptional changes modulating changes in synaptic plasticity, which, in turn, contribute to changes in behavior related to AUD, including alcohol reward, affective states, impulsivity, and cognitive function.

Therefore, targeting epigenetic regulators for the treatment of AUD and other addiction and neuropsychiatric disorders could be an exciting avenue for future investigations. Initial studies show promise in preclinical models, but these manipulations have not been widely explored in clinical populations. Multiple studies have shown that treatment with the class I/II HDAC inhibitor, Trichostatin A, can attenuate alcohol drinking and anxiety-related behaviors in rats and impact chromatin acetylation across several pertinent genes and brain regions [39,40,41,89,110]. Similarly, rats treated with suberoylanilide hydroxamic acid, an HDAC inhibitor clinically approved for the treatment of cutaneous T-cell lymphoma, showed decreased motivation to drink alcohol and alleviated depression-like behavior during alcohol withdrawal [34,47]. In clinical trials, valproic acid has shown some promise as a potential therapeutic for AUD, especially in managing withdrawal symptoms [117,118]. Compared to HDAC inhibitors, pharmacologically targeting HATs, especially in the brain, has proved to be more difficult and is still a largely unexplored domain. However, recently developed HAT activators are proving to have promising effects on increasing neurogenesis and improving long-term memory in rodents [119]. Other limitations, such as the limited translational validity of many preclinical alcohol exposure models and heterogeneity and comorbid diagnoses across AUD patients, pose additional questions on the translational potential of HDAC and HAT modulators across various patient populations. Altogether, pharmacologically targeting these epigenetic regulators is a promising therapeutic option, but more work is needed to develop agents that are organ and cell-type-specific.

Similarly, while the existence of sex differences in AUD is well established, the mechanisms underlying these differences are not well understood. Most studies that have investigated those mechanisms have focused on sex-specific hormones, particularly gonadal hormones, and their role in influencing the reinforcing effects of alcohol. While these studies are not without conflicting results [120], several studies reported correlations between estradiol and testosterone levels with alcohol intake and/or AUD status in women [121,122,123]. On the other hand, correlations between estradiol, follicle-stimulating hormone, testosterone, and progesterone levels with alcohol intake, cravings, and AUD status have been found in men [121,124,125]. In preclinical studies, high estrogen levels have been consistently associated with and contribute to higher alcohol drinking in female but not male rodents [120,126,127,128,129]. While the overall evidence supports the idea that sex differences in AUD may be driven by differences in gonadal hormones, there are many ways in which gonadal hormones could be acting within the brain to influence the behavioral response to alcohol, including nongenomic mechanisms [129]. Nevertheless, the best-known modes of action of steroidal hormones are as transcription factors and epigenetic modifiers, including leading to changes in histone modifications, which, in turn, could contribute to the observations described in this review. Therefore, more work is needed to understand how these functions contribute to individual and sex differences in propensity to develop excessive drinking and AUD.

The field of neuro-epigenetics has evolved quickly and is proving to be an ideal environment to uncover the molecular mechanisms of addiction and investigate novel treatment options. However, much of our current knowledge is based on studies that only utilized male rodents, used varied methods of alcohol delivery, and only investigated known transcriptional targets. There are many burgeoning techniques that add precision and granularity to epigenetic data, allowing researchers to further characterize the impact of alcohol on the chromatin landscape within single cells and specific cell types. Pairing sequencing with both widely utilized techniques, such as ChIP, and newer techniques, such as CUT&RUN, allows for a more precise interpretation of the results and may aid in identifying novel therapeutic targets through unbiased transcriptional profiling. Specifically, utilization of these techniques that isolate specific acetyl markers may uncover novel transcriptional targets, and an unbiased approach will allow for a wider understanding of the compilation of transcriptional changes associated with alcohol consummatory behaviors. In addition, this review primarily reports correlational results between alcohol exposure and histone acetylation or HAT/HDAC expression. Proving causality by directly manipulating these marks and their regulators will provide greater mechanistic validity and a deeper understanding of how the regulation of histone acetylation is directly related to alcohol use and addiction-related behaviors. With epigenetic mechanisms already being considered as targets for future treatment of addiction and neuropsychiatric disease, we desperately need to utilize all tools available to characterize the underlying mechanisms and identify novel areas of vulnerability. These efforts should be aided by direct comparisons between males and females across identical paradigms, and by using the most comprehensive and translationally relevant methods.

## Figures and Tables

**Table 1:** Alcohol’s effects on histone acetylation.

Brain Region	Histone Protein	Marker	Chronic/Acute	Route (Dose)	Withdrawal	Global/ Gene Specific (GS)	Species (Strain)	Sex	Refs
Cerebral Cortex	H3	H3K9/14ac	Acute (1 dose)	IP (3 g/kg)	6 h	Global: nc GS: ↓ GAD1, HDAC2	Mouse (C57BL/6J)	M	[21]
Cerebral hemisphere	H3	H3K9ac	Acute (1 dose)	Oral Gavage (6 g/kg)	1–12 h	Global: nc	Rat (Sprague Dawley)	M	[22]
Frontal Cortex	H3	H3K9ac	Chronic (8 doses)	IP (3 g/kg)	24 h	Global: nc	Rat (Wistar)	M	[23]
	H4	H4K12ac							
Prefrontal Cortex	H3	H3ac	Chronic (8 intermittent doses)	IP (3 g/kg)	24 h	GS: **↑** cFos and Bdnf (adolescent exposure): ↑ Bdnf (adult exposure)	Rat (Wistar)	M	[24]
		H3K9ac	Acute (2 doses 10 days apart)	IP (1.5–2.0 mg/kg)	24, 48 and 96 h	Global: nc	Mouse (DBA/2J)	M	[25]
			Chronic (various)	Various	0–96 h	Global: **↑** 0, 6 (subregions: IL and Lat.Orbital Cx) and 24 h; nc at 8, 48, 72, or 96 h	Mouse (C57BL/6J) Rat (Wistar)	M and F (mice)/M (rats)	[25,26,27,28]
					15 days–1 month	Global: nc; GS: ↑ GABA-Aα5	Mouse (C57Bl/6) Rat (Wistar)	M and F	[26,29,30]
		H3K14ac	Chronic (4 days)	IP (1 mg/kg) + Vapor (16 h)	0, 8 and 72 h	Global: nc	Mouse (C57BL/6J);	M	[28]
		H3K27ac	Chronic (4 days)	IP (1 mg/kg) + Vapor (16 h)	0, 8 and 72 h	Global: nc	Mouse (C57BL/6J);	M	[28]
	H4	H4ac	Chronic (8 doses)	IP (3 g/kg)	24 h	GS: ↑ FosB, Cdk5, Bdnf (adolescent exposure) **↓** FosB, Cdk5 (adult exposure)	Rat (Wistar)	M	[24]
					3 weeks	GS: ↑ fosb promoter, bdnf promoter	Mouse (C57Bl/6)	F	[26]
		H4K5ac	Chronic (8 doses)	IP (3 g/kg)	24 h and 3 weeks	Global: ↑	Mouse (C57Bl/6)	F	[26]
		H4K12ac	Chronic (8 doses)	IP (3 g/kg)	24 h and 3 weeks	Global: ↑ 24 h, nc 3 weeks	Mouse (C57Bl/6)	F	[26]
Striatum	H2A	H2Aac	Acute (1 dose)	IP (2.5 and 6 mg/kg)	1 h	Global: nc	Mouse (Swiss-Albino)	M	[31]
	H2B	H2Bac	Acute (1 dose)	IP (2.5 and 6 mg/kg)	1 h	Global: nc	Mouse (Swiss-Albino)	M	[31]
	H3	H3ac	Acute (1 dose)	IP (2.5 and 6 mg/kg)	1 h	Global: nc	Mouse (Swiss-Albino)	M	[31]
		H3K9ac	Acute (2 doses 10 days apart)	IP (1.5–2.0 mg/kg)	24, 48 and 96 h	Global: nc	Mouse (DBA/2J)	M	[25]
			Chronic (various)	IP (1.5–3 mg/kg) and Oral 2BC (12%)	24 h–15 days	Global: nc	Mouse (DBA/2J and C57Bl/6) Rat (Wistar)	M	[23,25,29]
		H3K14ac	Acute (1 dose)	IP (6 mg/kg)	1 h	Global: nc	Mouse (Swiss-Albino)	M	[31]
	H4	H4ac	Acute (1 dose)	IP (2.5 and 6 mg/kg)	1 h	Global: nc	Mouse (Swiss-Albino)	M	[31]
		H4K12ac	Acute (1 dose)	IP (2 g/kg)	30 min	Global: nc	Mouse (DBA/2J)	F	[32]
		H4K12ac	Chronic (various)	IP (2–3 g/kg)	30 min–24 h	Global: nc	Mouse (DBA/2J); Rat (Wistar)	F (mouse/M (Rat)	[23,32]
Nucleus Accumbens	H3	H3K9ac	Acute (2 doses 10 days apart)	IP (1.5–2.0 mg/kg)	24, 48 and 96 h	Global: nc in shell or core	Mouse (DBA/2J)	M	[25]
			Chronic (4 days)	IP (1 mg/kg) + Vapor (16 h)	0	Global: **↑**	Mouse (C57BL/6J)	M	[28]
			Chronic (various)	various	8–72 h	Global: nc	Mouse (C57BL/6J)/Rat (Wistar)	M	[23,28]
			Chronic (10 days)	IP (1.5–2.5 mg/kg)	24, 48 and 96 h	Global: **↑** in shell; nc in core	Mouse (DBA/2J)	M	[25]
		H3K14ac	Chronic (4 days)	IP (1 g/kg) + Vapor (16 h)	0 h	Global: **↑**	Mouse (C57BL/6J)	M	[28]
					8 and 72 h	Global: nc			
		H3K27ac	Acute (intermittent access 2 days)	Oral (10%)	0–22 h	Global nc at 0, 12, 18, and 22 h	Rat (Sprague Dawley)	Not specified	[33]
			Chronic (4 days)	IP (1 mg/kg) + Vapor (16 h)	0 h	Global: nc	Mouse (C57BL/6J)	M	[28]
					8 and 72 h	Global: nc			
			Chronic (10 days)	Oral (10% EtOH)	0–22 h	Global ↑ 12, 18, and 22 h; nc at 0 h	Rat (Sprague Dawley)	Not specified	[33]
	H4	H4ac	Chronic (intermittent access 8 weeks)	Oral 2BC + SA (20% EtOH)	0–1 h	Global: ↓	Mouse (C57Bl/6) Rat (Long-Evans)	M	[34]
		H4K12ac	Acute (1 dose)	IP (2 g/kg)	30 min	Global: **↑** (core); nc (shell)	Mouse (DBA/2J)	F	[32]
		H4K12ac	Chronic (10 doses)	IP (2 g/kg)	30 min	Global: **↑** (core); nc (shell)	Mouse (DBA/2J)	F	[32]
		H4K12ac	Chronic (8 intermittent doses)	IP (1 mg/kg); Vapor (16 h)	24 h	Global: nc	Rat (Wistar)	M	[23]
Basal Forebrain	H3	H3K14ac	Chronic (20 days)	Oral (6.8–Diet)	~24 h	Global: **↓**	Mouse (C57BL/6J)	M	[35]
Hypothalamus	H3	H3K9/14ac	Chronic 8 doses	IP (2 g/kg)	~50 days	GS: ↑ Mc4r promoters, Pomc promoters	Rat (Sprague Dawley)	M	[36]
		H3K14ac	Chronic (20 days)	Oral (6.8–Diet)	~24 h	Global: ↓	Mouse (C57BL/6J)	M	[35]
Amygdala	H3	H3K9/14ac	Acute (1 dose)	IP (1–2 g/kg)	1 h	GS: ↑ Hif3a, Creb1 proximal promoter, Cbp distal promoter, p300 proximal and distal promoters; nc Slc10a6	Rat (Sprague Dawley)	M	[37,38]
			Chronic AIE + Acute (1 dose)	IP (2 g/kg)	1 h	GS: ↑ Creb1 proximal promoter, Cbp proximal and distal promoters, p300 proximal and distal promoters	Rat (Sprague Dawley)	M	[38]
			Chronic (11 days)	Oral 2BC (9% EtOH)	0 h	GS: **↓** NPY promoter	Rat (P vs. NP)	M	[39]
			Chronic 8 doses (AIE)	IP (2 g/kg)	~50 days	GS: ↑ Mc4r promoters, ↓ NPY promoter	Rat (Sprague Dawley)	M	[36]
		H3K9ac	Acute (Various)	IP and Oral (1–1.5 g/kg)	1–24 h	Global: **↑** CeA and MeA; nc in BLA GS: **↑** pronociceptin and prodynorphin promoters	Rat (alcohol preferring (P) and SD)	M	[40,41,42]
			Chronic (various)	Oral (various) and Vapor	0 h	Global: ↑ CeA and MeA; nc BLA	Rat (P vs. NP)	M	[39]
					30 min	GS: ↑ prodynorphin promoter	Rat (Sprague Dawley)	M	[43]
					6 h	Global: ↑ CeA and BLA; nc in MeA	Rat (Wistar)	M	[27]
					24 h	Global: ↓ CeA and MeA; nc in BLA	Rat (Sprague Dawley)	M	[40]
					15 days	Global: nc	Mouse (C57Bl/6)	M	[29]
		H3K27ac	Acute (1 dose)	IP (1–2 g/kg)	1 h	GS: **↑** SARE and Arc promoter, Hif3a, and Slc10a6	Rat (Sprague Dawley)	M	[37,44]
			Chronic (2 doses every 2 days for 12 days)	IP (2 g/kg)	weeks	GS: ↓ Arc SARE site	Rat (Sprague Dawley)	M	[44]
			Chronic (individuals with AUD)	Oral (Individuals with AUD)	unknown	GS: ↓ ARC SARE site	Human	Mixed sex, but mostly male	[45]
	H4	H4K8ac	Acute (1 dose)	IP (1 g/kg)	1 and 24 h	Global: **↑** CeA and MeA; nc in BLA	Rat (Sprague Dawley)	M	[40,41]
			Chronic (10–16 days)	Oral (7–9%)	0 h	Global: ↑ CeA and MeA; nc in BLA; Global: nc in Sprague Dawley rats	Rat (P vs. NP; Sprague Dawley)	M	[39,40]
					24 h	Global: **↓** CeA and MeA; nc BLA	Rat (Sprague Dawley)	M	[40]
Hippocampus	H3	H3ac	Chronic (21 days)	Oral 2BC (9–10/mkg/kg/d)	0 h	Global: nc dentate gyrus and CA3 GS: Bdnf promoters: nc PII, **↓** PIII and PVIII; **↑** PVI	Mouse (C57BL/6J)	M	[46]
		H3K9/14ac	Acute (1 dose)	IP (3 g/kg)	6 h	Global: **↑**	Mouse (C57BL/6J)	M	[21]
		H3K9ac	Acute (1 dose)	IP (2 g/kg)	1 h	Global: **↑** GS: ↑ key neuronal genes	Mouse (C57BL/6J)	M	[13]
			Chronic (15 days–5 months)	Oral (12%)	0 h	Global: nc	Rat (Sprague Dawley)	M	[47]
					24 h	Global: **↓**			[47]
					15 days	Global: ↓	Mouse (C57Bl/6)	M	[29]
		H3K27ac	Acute (1 dose)	IP (2 g/kg)	1 h	Global: **↑** GS: ↑ key neuronal genes	Mouse (C57BL/6J)	M	[13]
	H4	H4ac	Chronic (21 days)	Oral 2BC (9–10 mg/kg)	0 h	Global: **↑** dentate gyrus; nc in CA3	Mouse (C57BL/6J)	M	[46]
		H4K12ac	Chronic (8 doses)	IP (3 g/kg)	24 h	Global: nc (0 h)	Rat (Wistar)	M	[23]
Lower Midbrain (including VTA)	H3	H3ac	Chronic (9 days)	Vapor (10 mL/dL)	0, 10 h and 3 days	Global: **↑** Global (0, 10 h and 3 d) GS: **↑** synaptic plasticity genes (10 h only)	Mouse (ddY)	M	[48]
	H4	H4ac	Chronic (9 days)	Vapor (10 mL/dL)	0, 10 h and 3 days	Global: **↑** Global (0, 10 h and 3 d) GS: nc	Mouse (ddY)	M	[48]

Abbreviations: 2BC: 2 bottle choice; AIE: adolescent intermittent ethanol; AUD: alcohol use disorder; BLA: basolateral amygdala; CeA: central amygdala; F: female; GS: gene specific; h: hours; IP: intraperitoneal; M: male; MeA: medial amygdala; min: minutes; nc: no change; NP: alcohol non-preferring; P: alcohol preferring; Refs: references; SA: self administration; VTA: ventral tegmental area.

**Table 3:** Alcohol’s effects on histone deacetylases.

Brain Region	Marker	Chronic/Acute	Route	Withdrawal	Effect	Species (Strain)	Sex	Refs
Cerebral Cortex	HDAC2	Acute (1 dose)	IP (3 g/kg)	6 h	mRNA: ↓	Mouse (C57BL/6J)	M	[21]
	HDAC11	Acute (1 dose)	IP (3 g/kg)	6 h	mRNA: ↓	Mouse (C57BL/6J)	M	[21]
Prefrontal Cortex	HDAC Activity	Acute (1 dose)	IP (2 g/kg)	30 min	enzymatic activity: ↑ (nuclear portion); nc (cytosol)	Mouse (DBA/2J)	F	[32]
		Chronic (8–10 doses)	IP (2–3 g/kg)	30 min	enzymatic activity: ↑ (nuclear portion); ↓ (cytosol)	Mouse (DBA/2J)	F	[32]
				24 h–2 weeks	enzymatic activity: nc	Rat (Wistar)	M	[24]
	HDAC1	Acute (1 dose)	IP (0.5–2 g/kg) & Oral (3 g/kg)	30 min–2 h	Protein & mRNA: nc	Rat (Sprague-Dawley & Wistar) Mouse (WT)	M (rats)/M & F (mice)	[106,107,108]
		Chronic (Various)	IP (0.5–2 g/kg) & Oral (3 g/kg)	30 min–2 h	mRNA: nc	Rat (Sprague-Dawley & Wistar)	M	[106,107]
	HDAC2	Acute (1 dose)	IP (0.5–2 g/kg) & Oral (3 g/kg)	30 min–2 h	Protein & mRNA: nc	Rat (Sprague-Dawley & Wistar) Mouse (WT)	M (rats)/M & F (mice)	[106,107,108]
		Chronic (Various)	IP (0.5–1 g/kg), EtOH Vapor, & Oral (3 g/kg)	0–2 h	mRNA: nc	Rat (Sprague-Dawley & Wistar) Mouse (C57Bl/6)	M	[28,106,107]
	HDAC3	Acute (1 dose)	IP (2 g/kg)	1 h	Protein: ↓ (nuclear portion); nc (cytosol)	Mouse (WT)	Mixed	[108]
			Oral: gavage (3 g/kg)	2 h	mRNA: nc	Rat (Wistar)	M	[107]
		Chronic (4 or 8 doses)	Oral: gavage (3 g/kg)	2 h	mRNA: nc	Rat (Wistar)	M	[107]
	HDAC4	Acute (1 dose)	IP (0.5 g/kg) & Oral (3 g/kg)	30 min–2 h	mRNA: nc	Rat (Sprague-Dawley & Wistar)	M	[106,107]
		Chronic (Various)	IP (0.5 g/kg)	30 min	mRNA:↓	Rat (Sprague-Dawley)	M	[106]
			Oral: gavage (3 g/kg)	2 h	mRNA: nc	Rat (Wistar)	M	[107]
	HDAC5	Acute (1 dose)	IP (0.5 g/kg) & Oral (3 g/kg)	30 min–2 h	mRNA: nc	Rat (Sprague-Dawley & Wistar)	M	[106,107]
		Chronic (Various)	IP (0.5 g/kg)	30 min	mRNA:↓	Rat (Sprague-Dawley)	M	[106]
			Oral: gavage (3 g/kg)	2 h	mRNA: nc	Rat (Wistar)	M	[107]
	HDAC6	Acute (1 dose)	Oral: gavage (3 g/kg)	2 h	mRNA: nc	Rat (Wistar)	M	[107]
		Chronic (4 or 8 doses)						
	HDAC7	Acute (1 dose)	Oral: gavage (3 g/kg)	2 h	mRNA: nc	Rat (Wistar)	M	[107]
		Chronic (4 or 8 doses)						
	HDAC8	Acute (1 dose)	Oral: gavage (3 g/kg)	2 h	mRNA: nc	Rat (Wistar)	M	[107]
		Chronic (4 or 8 doses)						
	HDAC9	Acute (1 dose)	Oral: gavage (3 g/kg)	2 h	mRNA: nc	Rat (Wistar)	M	[107]
		Chronic (4 or 8 doses)						
	HDAC10	Acute (1 dose)	Oral: gavage (3 g/kg)	2 h	mRNA: ↑	Rat (Wistar)	M	[107]
		Chronic (4 or 8 doses)	Oral: gavage (3 g/kg)	2 h	mRNA: nc	Rat (Wistar)	M	[107]
	HDAC11	Acute (1 dose)	Oral: gavage (3 g/kg)	2 h	mRNA: nc	Rat (Wistar)	M	[107]
		Chronic (Various)	IP (1 g/kg), EtOH Vapor, & Oral (3 g/kg)	0–2 h	mRNA: nc	Mouse (C57BL/6J) Rat (Wistar)	M	[28,107]
Striatum	HDAC Activity	Acute (1 dose)	IP (2 g/kg)	30 min	enzymatic activity: ↓ (nuclear portion); ↑ (cytosol)	Mouse (DBA/2J)	F	[32]
		Chronic (10 doses)						
	HDAC1	Acute (1 dose)	IP (0.5–2 g/kg)	30 min	mRNA: nc (dorsal and whole striatum)	Mouse (DBA/2J) Rat (Sprague-Dawley)	F (mice)/M (rats)	[32,106]
				1 h	Protein: ↓ (nuclear portion, dorsal striatum)	Mouse (WT)	Mixed	[108]
		Chronic (Various)	IP (0.5–2 g/kg)	30 min	mRNA: nc (dorsal and whole striatum)	Mouse (DBA/2J) Rat (Sprage-Dawley)	F (mice)/M (rats)	[32,106]
	HDAC2	Acute (1 dose)	IP (0.5 g/kg)	30 min	mRNA: nc (dorsal striatum)	Rat (Sprague-Dawley)	M	[106]
			IP (2 g/kg)	30 min	mRNA: ↓	Mouse (DBA/2J)	F	[32]
			IP (2 g/kg)	1 h	Protein: ↓ (nuclear portion) nc (cytosol) (dorsal striatum)	Mouse (WT)	Mixed	[108]
		Chronic (Various)	IP (0.5 g/kg)	30 min	mRNA: nc (dorsal striatum)	Rat (Sprague-Dawley)	M	[106]
			IP (2 g/kg)	30 min	mRNA: ↓	Mouse (DBA/2J)	F	[32]
	HDAC3	Acute (1 dose)	IP (2 g/kg)	30 min	mRNA: nc	Mouse (DBA/2J)	F	[32]
				1 h	Protein: ↓ (nuclear portion); nc (cytosol) (dorsal striatum)	Mouse (WT)	Mixed	[108]
		Chronic (10 doses)	IP (2 g/kg)	30 min	mRNA: nc	Mouse (DBA/2J)	F	[32]
	HDAC4	Acute (1 dose)	IP (0.5–2 g/kg)	30 min	mRNA: nc (dorsal and whole striatum)	Mouse (DBA/2J) Rat (Sprage-Dawley)	F (mice)/M (rats)	[32,106]
		Chronic (Various)	IP (0.5 g/kg)	30 min	mRNA: ↓ (dorsal striatum)	Rat (Sprague-Dawley)	M	[106]
			IP (2 g/kg)	30 min	mRNA: nc	Mouse (DBA/2J)	F	[32]
	HDAC5	Acute (1 dose)	IP (0.5–2 g/kg)	30 min	mRNA: nc (dorsal and whole striatum)	Mouse (DBA/2J) Rat (Sprage-Dawley)	F (mice)/M (rats)	[32,106]
		Chronic (Various)	IP (0.5 g/kg)	30 min	mRNA: ↓ (dorsal striatum)	Rat (Sprague-Dawley)	M	[106]
			IP (2 g/kg)	30 min	mRNA: nc	Mouse (DBA/2J)	F	[32]
	HDAC6	Acute (1 dose)	IP (2 g/kg)	30 min	mRNA: nc	Mouse (DBA/2J)	F	[32]
		Chronic (10 doses)						
	HDAC7	Acute (1 dose)	IP (2 g/kg)	30 min	mRNA: nc	Mouse (DBA/2J)	F	[32]
		Chronic (10 doses)	IP (2 g/kg)	30 min	mRNA: ↑	Mouse (DBA/2J)	F	[32]
	HDAC8	Acute (1 dose)	IP (2 g/kg)	30 min	mRNA: ↑	Mouse (DBA/2J)	F	[32]
		Chronic (10 doses)	IP (2 g/kg)	30 min	mRNA: nc	Mouse (DBA/2J)	F	[32]
	HDAC9	Acute (1 dose)	IP (2 g/kg)	30 min	mRNA: ↑	Mouse (DBA/2J)	F	[32]
		Chronic (10 doses)	IP (2 g/kg)	30 min	mRNA: nc	Mouse (DBA/2J)	F	[32]
	HDAC10	Acute (1 dose)	IP (2 g/kg)	30 min	mRNA: nc	Mouse (DBA/2J)	F	[32]
		Chronic (10 doses)	IP (2 g/kg)	30 min	mRNA: ↓	Mouse (DBA/2J)	F	[32]
	HDAC11	Acute (1 dose)	IP (2 g/kg)	30 min	mRNA: ↓	Mouse (DBA/2J)	F	[32]
		Chronic (10 doses)	IP (2 g/kg)	30 min	mRNA: ↓	Mouse (DBA/2J)	F	[32]
Nucleus Accumbens	HDAC Activity	Acute (2 days)	Oral (10% EtOH)	0–22 h	Activity: (nuclear) nc at 0, 12, 18 or 22 h	Rat (Sprague-Dawley)	Unknown	[33]
		Chronic (10 days)	Oral (10% EtOH)	0–22 h	Activity: (nuclear) ↓ at 22 h, nc at 0, 12, or 18 h	Rat (Sprague-Dawley)	Unknown	[33]
	HDAC2	Chronic (4 doses)	IP (1 g/kg) + EtOH vapor	0 h	mRNA: nc	Mouse (C57BL/6J)	M	[28]
	HDAC4	Chronic (2 h/day × 10 days)	Oral (10% EtOH)	0–22 h	Protein: (nuclear) ↓ 18 and 22 h; nc at 0 or 12 h; (cytoplasm as measured by mRNA) nc at 0, 12, 18, or 22 h	Rat (Sprague-Dawley)	Unknown	[33]
	HDAC11	Chronic (Various)	IP (1 g/kg) + EtOH vapor	0 h	mRNA: nc	Mouse (C57BL/6J)	M	[28]
			Oral 2BC (10% EtOH)	6 days	mRNA: ↑	Mouse (C57Bl/6NCrl)	M	[109]
Amygdala	HDAC Activity	Acute (1 dose)	IP (1g/kg)	1–3 h	enzymatic activity: ↓	Rat (Sprague Dawley, P & NP)	M	[40,41,42]
			IP (2 g/kg)	30 min	enzymatic activity: nc	Mouse (DBA/2J)	F	[32]
		Chronic (Various)	IP (2 g/kg) and Oral: 9% *v*/*v* Lieber-Decarli liquid diet	0–30 min	enzymatic activity: nc	Mouse (DBA/2J) Rat (Sprague-Dawley)	F (mice)/M (rats)	[32,40]
				1 h	enzymatic activity: ↓ (nuclear portion); nc (cytosol)	Rat (Sprague Dawley)	M	[110]
				24 h	enzymatic activity: ↑ (CeA & MeA); nc (BLA)	Rat (Sprague Dawley)	M	[40,110]
				52 days	enzymatic activity: ↑ (nuclear portion); nc (cytosol)	Rat (Sprague Dawley)	M	[110]
	HDAC1	Acute (1 dose)	Oral: gavage (3 g/kg)	2 h	mRNA: ↑	Rat (Wistar)	M	[107]
		Chronic (4 or 8 doses)	Oral: gavage (3 g/kg)	2 h	mRNA: nc	Rat (Wistar)	M	[107]
	HDAC2	Acute (1 dose)	IP (1 g/kg)	1 h	Protein: ↓ (MeA & CeA); nc (BLA)	Rat (P)	M	[42]
			Oral: gavage (3 g/kg)	2 h	mRNA: ↑	Rat (Wistar)	M	[107]
		Chronic (Various)	IP (2 g/kg) and Oral (various)	0 h	Protein: ↓	Rat (P & NP)	M	[39]
				1–2 h	Protein & mRNA: nc	Rat (Sprague Dawley, Wistar)	M	[107,110]
				24 h–52 days	Protein & mRNA: ↑ (CeA & MeA); nc (BLA)	Rat (Sprague Dawley)	M	[110]
	HDAC3	Acute (1 dose)	Oral: gavage (3 g/kg)	2 h	mRNA: nc	Rat (Wistar)	M	[107]
		Chronic (4 or 8 doses)						
	HDAC4	Acute (1 dose)	IP (1g/kg) & Oral (3 g/kg)	1–2 h	Protein & mRNA: nc	Rat (Wistar, P & NP)	M	[42,107]
		Chronic (4 or 8 doses)	IP (2 g/kg) & Oral (3 g/kg)	1–2 h	Protein & mRNA: nc	Rat (Sprague Dawley, Wistar)	M	[107,110]
				24 h	Protein: ↑ (CeA & MeA); nc (BLA)	Rat (Sprague Dawley)	M	[110]
				52 days	Protein: nc	Rat (Sprague Dawley)	M	[110]
	HDAC5	Acute (1 dose)	Oral: gavage (3 g/kg)	2 h	mRNA: ↑	Rat (Wistar)	M	[107]
		Chronic (4 or 8 doses)	Oral: gavage (3 g/kg)	2 h	mRNA: nc	Rat (Wistar)	M	[107]
	HDAC6	Acute (1 dose)	Oral: gavage (3 g/kg)	2 h	mRNA: nc	Rat (Wistar)	M	[107]
		Chronic (4 or 8 doses)						
	HDAC7	Acute (1 dose)	Oral: gavage (3 g/kg)	2 h	mRNA: nc	Rat (Wistar)	M	[107]
		Chronic (4 or 8 doses)						
	HDAC8	Acute (1 dose)	Oral: gavage (3 g/kg)	2 h	mRNA: nc	Rat (Wistar)	M	[107]
		Chronic (8 doses)	Oral: gavage (3 g/kg)	2 h	mRNA: ↑	Rat (Wistar)	M	[107]
	HDAC9	Acute (1 dose)	Oral: gavage (3 g/kg)	2 h	mRNA: nc	Rat (Wistar)	M	[107]
		Chronic (4 or 8 doses)						
	HDAC10	Acute (1 dose)	Oral: gavage (3 g/kg)	2 h	mRNA: nc	Rat (Wistar)	M	[107]
		Chronic (4 or 8 doses)						
	HDAC11	Acute (1 dose)	Oral: gavage (3 g/kg)	2 h	mRNA: nc	Rat (Wistar)	M	[107]
		Chronic (4 or 8 doses)						
Hippocampus	HDAC Activity	Acute (1 dose)	IP 1 dose 2 g/kg	30 min	enzymatic activity: nc	Mouse (DBA/2J)	F	[32]
		Chronic (8–10 doses)	IP (2 g/kg)	30 min	enzymatic activity: nc	Mouse (DBA/2J)	F	[32]
				52 days	enzymatic activity: ↑ (nuclear portion); nc (cytosol)	Rat (Sprague Dawley)	M	[83]
	HDAC1	Chronic (21 days)	Oral: Avg 9–10 g/kg per day	0 h	mRNA: ↓	Mouse (C57BL/6J)	M	[46]
	HDAC2	Acute (1 dose)	IP (3 g/kg)	7 h	mRNA: nc	Mouse (C57BL/6J)	M	[21]
		Chronic (15–21 days)	Oral: 10% *v*/*v*	0 h	mRNA: ↓	Mouse (C57BL/6J)	M	[46]
			Oral: 9% *v*/*v* Lieber-Decarli liquid diet	0 h	mRNA: ↑	Rat (Sprague Dawley)	M	[47]
				24 h	mRNA: ↑	Rat (Sprague Dawley)	M	[47]
	HDAC3	Chronic (21 days)	Oral: 10% *v*/*v*	0 h	mRNA: nc	Mouse (C57BL/6J)	M	[46]
	HDAC4	Chronic (21 days)	Oral: 10% *v*/*v*	0 h	mRNA: nc	Mouse (C57BL/6J)	M	[46]
	HDAC5	Chronic (21 days)	Oral: 10% *v*/*v*	0 h	mRNA: ↓	Mouse (C57BL/6J)	M	[46]
	HDAC9	Chronic (21 days)	Oral: 10% *v*/*v*	0 h	mRNA: nc	Mouse (C57BL/6J)	M	[46]
	HDAC11	Acute (1 dose)	IP (3 g/kg)	6 h	mRNA: nc	Mouse (C57BL/6J)	M	[21]

Abbreviations: 2BC: 2 bottle choice; BLA: basolateral amygdala; CeA: central amygdala; F: female; HDAC: histone deacetylase; h: hours; IP: intraperitoneal; M: male; MeA: medial amygdala; min: minutes; nc: no change; NP: alcohol non-preferring; P: alcohol preferring; Refs: references; VTA: ventral tegmental area.

## Data Availability

No new data were created or analyzed in this study.
